# Comparison of C-Reactive Protein in Dried Blood Spots and Saliva of Healthy Adolescents

**DOI:** 10.3389/fimmu.2021.795580

**Published:** 2021-12-16

**Authors:** Anne-Christine Plank, Janina Maschke, Nicolas Rohleder, Peter A. Fasching, Matthias W. Beckmann, Johannes Kornhuber, Anna Eichler, Gunther H. Moll, Oliver Kratz

**Affiliations:** ^1^ Department of Child and Adolescent Mental Health, University Hospital Erlangen, Friedrich-Alexander-University Erlangen-Nürnberg, Erlangen, Germany; ^2^ Department of Psychology, Friedrich-Alexander-University Erlangen-Nürnberg, Erlangen, Germany; ^3^ Department of Obstetrics and Gynecology, University Hospital Erlangen, Friedrich-Alexander-University Erlangen-Nürnberg, Erlangen, Germany; ^4^ Department of Psychiatry and Psychotherapy, University Hospital Erlangen, Friedrich-Alexander-University Erlangen-Nürnberg, Erlangen, Germany

**Keywords:** C-reactive protein, CRP, dried blood spots, DBS, saliva, adolescents, inflammation, BMI

## Abstract

**Background/Aim:**

Determining C-reactive protein (CRP) by non-invasive methods is of great interest for research addressing inflammation in young people. However, direct comparisons of such methods applied in children and adolescents are lacking so far. This study aimed to evaluate the association between CRP measured in dried blood spots (DBS CRP) and in saliva (sCRP), two less invasive alternatives to venipuncture, in 12- to 14-year-old adolescents. To evaluate the validity of both measurements in the context of biobehavioral studies, the potential of DBS CRP and sCRP to discriminate between defined BMI subgroups was assessed.

**Materials and Methods:**

CRP levels in DBS and saliva collected from 87 healthy adolescents (*M* = 13.25 years, *SD* = 0.30, 51.7% females) were determined using high sensitive CRP ELISA for serum and salivary CRP ELISA, respectively. Characteristics and correlation of both measurements were assessed for the total sample and for three subgroups classified by BMI percentile ranges (A: ≤ 25; B: 26–74; C: ≥ 75).

**Results:**

In the total sample, DBS CRP and sCRP were significantly associated (*r* = 0.59, *p* < 0.001). Splitting the sample into BMI-dependent subgroups revealed similarly strong associations of DBS CRP with sCRP for all three groups (A: *r* = 0.51; B: *r* = 0.61; C: *r* = 0.53). However, comparing the mean CRP values per BMI subgroup, one-way ANOVA reported significant differences for DBS CRP, but not for sCRP mean values.

**Conclusions:**

The significant correlation of DBS CRP with sCRP was independent of the investigated BMI range groups, yet BMI-dependent distinction was only provided by DBS CRP mean values. Overall, our results suggest that DBS CRP is likely to reflect systemic inflammation more precisely. Salivary CRP can be alternatively determined in studies with adolescents when conditions require it, given the oral health status is assessed. Considering that DBS CRP and sCRP share only 35% of common variance, further studies should examine their specific validity.

## 1 Introduction

The assessment of inflammatory markers is an important tool in everyday clinical practice. C-reactive protein (CRP) is an acute-phase reactant of the innate immune system, which activates the classical complement pathway and promotes phagocytosis. Its secretion by the liver is triggered *via* interleukin 6 in response to acute inflammation or injury (1, 2). Hence, CRP is assessed clinically to diagnose and monitor inflammatory conditions, such as infections and trauma, or systemic autoimmune disease (3). While high levels of CRP are related to an acute inflammatory response, mildly increased circulating CRP is, among others, associated with genetic factors or subclinical inflammatory processes occurring in the context of obesity and other conditions (1, 2, 4, 5). Such moderately elevated levels of baseline CRP, determined *via* high-sensitivity assays, are a predictor of cardiovascular disease, with CRP levels > 3 mg/L indicating a high risk of future cardiovascular events (6, 7). Since evidence suggests a relationship of psychosocial stress and psychiatric disorders with low-grade systemic inflammation (8), CRP levels are examined by a growing body of research in this field. A variety of studies confirms an association of elevated CRP with depressive symptoms (9–13), post-traumatic stress disorder (PTSD) (14, 15), and psychosis (16–18) as well as with perceived psychosocial stress (19) in adults. Positive affect, in turn, is considered to be negatively associated with CRP and to buffer against stress-related increases in CRP levels (20, 21). Regarding children and adolescents, a recent meta-analysis reports on a positive association between CRP and depressive symptoms (22), and elevated CRP has also been observed in children exposed to different psychosocial stressors (23–25). A study investigating the effects of early life adversity, which is related to elevated CRP levels in adulthood (25), found an association between elevated salivary CRP and psychosocial stress in infants (26). Similarly, intrauterine alcohol exposure has recently been linked to elevated DBS CRP levels (27).

For clinical diagnostic and monitoring purposes, CRP is commonly measured in venous blood. However, since the procedure of venipuncture is invasive and requires a medical setting with trained personnel, it is not always practicable in biobehavioral studies, especially in those investigating stress-sensitive outcomes and dealing with young participants. Here, measuring CRP in saliva or in a dried spot of capillary blood obtained from a finger prick could be more suitable alternatives. Both methods have been established and validated over the past years, and CRP determined in capillary dried blood spots (DBS) has been shown to strongly correlate with CRP levels in venous blood samples (28, 29). In contrast to the latter, DBS specimens can be collected by non-medical personnel and do not require special equipment or immediate freezing for storage: the filter paper soaked with a few drops of blood can be dried and stored at controlled ambient temperature for several hours (29). Yet, even though pricking a finger with a lancet is much less invasive than venipuncture, it may still cause stress or discomfort in some subjects, especially in children or infants.

These issues can be avoided by analyzing saliva samples, whose collection is painless, stress-free, rapid, and easy to perform by non-medical staff or the participant, optionally at home and multiple times. Consequently, validating the diagnostic potential of biomarker levels in saliva is of great interest. To date, numerous studies comparing salivary CRP (sCRP) with blood CRP levels have found moderate to strong correlations in neonates, adolescents, and adults (30–34). However, Dillon and colleagues (35) could not establish any association between both parameters in healthy adults, and an article reviewing correlations reported by 14 studies concludes that the overall association of sCRP with serum CRP is moderate (36). The level of inflammatory biomarkers in oral fluid can be influenced by different local factors, comprising oral diseases, injuries, and hygiene as well as salivary flow rate and regulatory mechanisms (36–38). These aspects might account for a seemingly less robust association of sCRP—compared to DBS CRP—with serum CRP and must be considered whenever saliva specimens are analyzed.

Overall, both approaches of non-invasively measuring CRP have certain advantages and disadvantages, yet to our knowledge, there is only one study to date that has directly evaluated the association between DBS CRP and sCRP levels. Goetz and Lucas (39) measured the CRP response to acute social stress in both specimens, collected from adult African-Americans at different stages of a social-evaluative stressor task. They report on a modestly positive correlation at baseline conditions (*r* = 0.183, *p* = 0.059) and a stress-related increase in CRP, which was only detected in saliva (39). For this type of stress research, oral fluid analysis appears beneficial, as both repeated measurements and a minimally invasive, non-stress-provoking sampling procedure are required. The latter is also desirable in studies with children or adolescents, taking into account a potential discomfort at the sight of blood.

## 2 Objective

In the present study, we aimed to extend the comparative data on DBS CRP and sCRP to this group of subjects and asked for the relationship of the two measurements in healthy adolescents. First, we examined the correlation of DBS CRP with sCRP in the total sample, controlling for potential and known confounders. Since the body mass index (BMI) is a correlate of systemic inflammation and has been demonstrated to be positively associated with CRP levels (5, 30, 32, 40, 41), we split our sample into three BMI-dependent subgroups and determined the association of DBS CRP and sCRP within these groups. To evaluate the validity of both measurements in the context of biobehavioral studies, we finally assessed the potential of both measurements to discriminate between the BMI subgroups.

## 3 Materials and Methods

### 3.1 Study Design and Participants

The present study is based on data collected in the Franconian Cognition and Emotion Studies (FRANCES) (42, 43), a follow-up study of the prospective longitudinal Franconian Maternal Health Evaluation Studies (FRAMES) (44, 45). From the outpatient inflow at the Department of Obstetrics and Gynecology *n* = 1100 women were recruited during their third trimester of pregnancy (FRAMES). Between 2012 and 2015, when children attended primary school, a subsample of these women (*n* = 618) was contacted for re-participation. Finally, *n* = 245 FRAMES mother–child dyads (39.6%; child age: *M* = 7.74, *SD* = 0.74, range = 6.00–9.90) agreed to take part in the FRANCES I follow-up wave. The participating women did not differ from the non-participating women in marital status [*χ^2^* (1) = 0.16, *p* = 0.690], educational level [*χ^2^* (1) = 0.08, *p* = 0.774], or family income [*χ^2^* (2) = 0.97, *p* = 0.616] at time of childbirth (FRAMES). Mothers and children participating in FRANCES I were contacted again from 2019 to 2021 to take part in the second follow-up wave, FRANCES II (46). Of the 245 contacted families, 186 (75.9%) with *n* = 188 children (due to two pairs of twins) agreed to participate again (child age: *M* = 13.3, *SD* = 0.34, range 12.8–14.4), of which 167 children (89.8%) participated in person (two 2-h data collection sessions on two different days, including physical examination, neuropsychological testing, interview, questionnaires, and biomarker sample collection) and 21 children (10.2%) only filled out questionnaires by post. When comparing participating families with non-participating families, no differences in marital status [*χ^2^* (1) = 0.35, *p* = 0.552], family income [*χ^2^* (4) = 3.94, *p* = 0.414], or maternal total psychopathology [*t*(234) = −0.93, *p* = 0.353] at time of FRANCES I were found.

In the present study, CRP levels of FRANCES II adolescents participating in person were analyzed. A total of *n* = 167 youths were eligible, with the following *n* = 62 (37.1%) drop-outs: *n* = 1 no consent to any sample collection; *n* = 1 no consent to saliva collection; *n* = 12 no consent to DBS collection; *n* = 6 DBS too small for analysis; *n* = 24 no saliva sample due to Corona-pandemic; *n* = 4 insufficient saliva sample quality; *n* = 4 anti-inflammatory medication on the day of sampling; and *n* = 10 oral injury/inflammation. Oral health was assessed *via* a questionnaire that included the following items: current oral injuries, oral inflammation, oral tumors (yes - no, providing further details if yes). Specifically, the following cases were excluded: gingivitis (*n* = 3), injury due to braces (*n* = 3), bitten on the cheek (*n* = 2), bitten on the lip (*n* = 1), and blister in mouth (*n* = 1). Accordingly, the analyses are based on *n* = 105 participants, of which DBS CRP and sCRP were present. None of these participants reported severe health issues (acute infectious, malignant, endocrine, or autoimmune disorders), intake of β-blockers or systemic steroid-based anti-inflammatory medication, alcohol consumption, or smoking. *N* = 18 further exclusions, due to CRP measurement aspects, are described in the following sections.

The study protocol was authorized by the Local Ethics Committee of the Faculty of Medicine at the University of Erlangen-Nürnberg and was performed in line with the Declaration of Helsinki. All mothers gave written consent for the research and the publication of the results; in addition, all children gave informed assent.

### 3.2 Specimen Collection

#### 3.2.1 DBS

At the end of a 1.5-h neuropsychological test session, a lancet (Safety-Lancet Extra 18G, penetration depth 1.8 mm, Sarstedt, Nümbrecht, Germany) was used for pricking the participant’s finger after cleansing it with 70% EtOH to collect at least one drop of capillary blood. The blood drops were applied to specimen collection paper (903 Protein Saver Snap Apart Cards, Whatman, GE Healthcare, Cardiff, UK). After at least 8 h of drying at room temperature, the specimens were packed into sealed bags (Multi Barrier Pouches, Whatman, GE Healthcare, Cardiff, UK) together with a 0.5-g silica gel sachet (Celloexpress, Antrim, UK) and stored at −80°C until analysis.

#### 3.2.2 Saliva

Saliva was also collected at the end of the 1.5-h neuropsychological test session, during which participants did not eat, drink, chew gum, or smoke. The participant was instructed to passively collect saliva in the mouth and release it into a sampling tube *via* a piece of straw (SaliCap Set, IBL International, Hamburg, Germany). Samples were kept on ice until preliminary processing within 0.5 h of collection: Saliva was centrifuged at 20,000 × *g* at 4°C for 2 min to remove cells and mucus. The collected supernatant was then aliquoted and stored at −80°C until analysis. Samples with visible signs of blood contamination were excluded from analysis.

### 3.3 CRP Measurement

#### 3.3.1 DBS

A CRP high-sensitive ELISA (hsCRP-ELISA; IBL International, Hamburg, Germany) for human serum and plasma was used for the quantitative determination of CRP concentrations in DBS eluate (DBS CRP). Sample preparation was performed as described by Danese and colleagues (23). First, a 3.5-mm core of the blood spot was punched out of the filter paper using a Biopunch^®^ hand punch (Plano, Wetzlar, Germany) and each sample was transferred into one well of a 96-well plate (Sarstedt, Nümbrecht, Germany). After an overnight elution at 4°C in 250 μl of phosphate buffered saline (Roth, Karlsruhe, Germany) containing a protease inhibitor cocktail (cOmplete mini, Roche, Basel, Switzerland) and 0.1% Tween 20 (Roth, Karlsruhe, Germany), samples were incubated for 1 h at room temperature on a microplate shaker (300 rpm).

CRP levels in 100 µl of undiluted DBS eluate and in standard sera diluted 1:1,000 were measured following the manufacturer’s protocol. Optical density was determined at 450 nm using the Benchmark Plus Microplate Reader (BioRad, Hercules, California, USA); the samples’ CRP levels were then quantified against a standard curve generated *via* a five-parameter logistic curve fit. Each sample, calibrator, and control was assayed in duplicate, and the average of the duplicate was used for statistical analyses. The intra- and inter-assay coefficient of variation (CV) was 6.9% and 6.3%, respectively; analytical sensitivity was approximately 0.02 μg/ml.

#### 3.3.2 Saliva

The concentration of CRP in saliva (sCRP) was determined using a commercially available ELISA kit (Salivary C-Reactive Protein ELISA Kit Generation II, Salimetrics Inc., State College, PA, USA) following the manufacturer’s instructions. Briefly, samples were thawed to room temperature on the day of analysis and centrifuged at 1,500 × *g* for 15 min to remove any remaining contaminants. Optical density was determined at 450 nm using the Benchmark Plus Microplate Reader (BioRad, Hercules, California, USA). The samples’ CRP levels were then quantified against a standard curve generated *via* a four-parameter logistic curve fit. Each sample, calibrator, and control was assayed in duplicate, and the average of the duplicate was used in statistical analyses. The intra- and inter-assay CV was less than 10%. The lower limit of detection, defined as the minimal detectable concentration, was 0.042 pg/ml (analytical sensitivity), and the lower limit of quantification was 19.44 pg/ml (functional sensitivity).

### 3.4 Data Analysis and Statistics

Of the 105 pairs of analyzed samples, 16 were excluded because the CV of one of the respective sample duplicate measurements exceeded 20%. Outliers with CRP values exceeding four standard deviations above the mean of the respective measurement were excluded as well (*n* = 2), resulting in an effective sample size of *n* = 87 sample pairs.

#### 3.4.1 Potential Covariates

The following variables were considered as potential covariates: age; sex (assigned at birth); BMI [calculated from weight and height measured in FRANCES II (kg/m^2^) and presented as percentiles accounting for sex and age (47)]; regular medication intake during the past 6 months prior to DBS and saliva sampling (for the analyzed sample, medication intake reported by the mother comprised hormonal contraception, topical/inhaled steroids, antihistamines, antibiotics and antifungal medication); and socioeconomic status [SES, calculated as sum of scores for education of both parents (4 = 12–13 years of schooling; 3 = 10 years of schooling; 2 = 9 years of schooling; 1 = <9 years of schooling) and family income (six levels: <1,000 Euro/month to >5,000 Euro/month); sum-index theoretical range: 3–14].

#### 3.4.2 Statistical Analysis

Statistical analyses were performed using IBM SPSS Statistics software (Version 24.0, IBM, Armonk, NY, USA). Since the Shapiro–Wilk test for normality revealed that both DBS CRP and sCRP levels lacked normal distribution [DBS CRP: *W*(87) = 0.639, *p* < 0.001; sCRP: *W*(87) = 0.741, *p* < 0.001], logarithmically transformed (log10) values were used for further analyses. Means (*M*), standard deviations (*SD*), and frequencies (*n*) with percentages (%) are reported for CRP values and sample characteristics. Potential covariates were tested using Pearson’s correlations (*r*) (BMI, age, SES) or *t*-tests (*t*)/Mann–Whitney tests (*U*; in case of subgroup *n* < 10) for independent samples (sex/medication intake yes vs. no). Homogeneity of variances was assessed *via* Levene’s test. The relationship between DBS CRP and sCRP levels was evaluated *via* Pearson’s correlation (*r*) and partial correlation (*r_p_*) controlling potential covariates. Outcomes with |r| ≥ .10 were interpreted as weak, |r| ≥ .30 were interpreted as mildly associated, and |r| ≥ .50 were interpreted as strongly correlated (48). For BMI-dependent analyses, the sample was divided into three subgroups (A: BMI percentile ≤ 25, B: BMI percentile 26–74, C: BMI percentile ≥ 75). To compare the level of correlations of DBS CRP and sCRP per BMI-subgroup, the correlation coefficients *r* were converted to *z_r_ via *Fisher transformation, and a *z*-score of the differences between the correlations was calculated (*Z_difference_*). Mean values of DBS CRP/sCRP per BMI subgroup were compared *via* one-way analysis of variance (ANOVA), reporting *F*-values, *p*-values, and partial eta squared (*η* ^2^) for effect size estimations [*η* ^2^ ≥ .01 can be interpreted as small effect, *η* ^2^ ≥ .06 represents a medium effect size, and *η* ^2^ ≥ .14 is interpreted as a strong effect (48)]. Hochberg’s GT2 was used for *post-hoc* testing (based on confirmed homogeneity of population variances), if appropriate. For graphical depiction, DBS CRP log10 and sCRP log10 means per subgroup were *z*-transformed. The level of significance was defined as *p* < 0.05 (two-tailed).

## 4 Results

### 4.1 Descriptive Data

Descriptive statistics of the sample’s characteristics are presented in **Table 1**. The sample comprised 45 (51.7%) girls and 42 (48.3%) boys. There were no sex-dependent BMI-percentile differences [*t*(85) = −1.45, *p* = 0.152].

**Table 1:** Descriptive data.

Variable	*n*	*mean (SD)*	*min*	*max*
DBS CRP (mg/L)	87	0.97 (1.43)	0.02	8.64
DBS CRP (log10)	87	−0.37 (0.59)	−1.81	0.94
DBS CRP (log10) - girls	45	−0.30 (0.54)	−1.28	0.94
DBS CRP (log10) - boys	42	−0.46 (0.64)	−1.81	0.78
sCRP (pg/ml)	87	155.63 (159.28)	18.34	722.15
sCRP (log10)	87	2.02 (0.39)	1.26	2.86
sCRP (log10) - girls	45	2.03 (0.40)	1.26	2.86
sCRP (log10) - boys	42	2.00 (0.38)	1.36	2.81
Age	87	13.25 (0.30)	12.78	14.38
SES index (range 3–14)	87	11.98 (1.82)	8.00	14.00
BMI (percentile)	87	50.28 (28.46)	0.00	97.00

DBS CRP, CRP measured in dried blood spot eluate; sCRP, salivary CRP; SES, socioeconomic status, calculated as sum of scores for parental education of both parents (4 = 12-13 years of schooling; 3 = 10 years of schooling; 2 = 9 years of schooling; 1 = < 9 years of schooling) and family income (6-levels: < 1000 Euro/month to > 5000 Euro/month) (sum-index, theoretical range: 3–14); BMI, body mass index.

### 4.2 Potential Covariates

Testing of potential covariates revealed that neither SES, age, and sex (*n* = 87), nor medication intake during the 6 months prior to sampling (yes: *n* = 8) were significantly associated with CRP levels measured in DBS eluate and saliva (**Table 2**). However, a significant positive correlation of the BMI with DBS CRP (*r* = 0.40, *p* < 0.001) and sCRP (*r* = 0.28, *p* = 0.009) was detected.

**Table 2:** Correlations and mean comparisons of DBS CRP/sCRP levels and potential confounders.

Potential confounders	DBS CRP (log10)		sCRP (log10)	
	*r*	*p*	*r*	*p*
Age	−0.13	0.225	−0.05	0.670
SES	0.07	0.538	0.02	0.884
BMI	0.40	<0.001**	0.28	0.009**
	***t (df)/U***	***p***	***t (df)/U***	***p***
Sex	−1.248 (85)	0.215	−0.291 (85)	0.772
Medication intake	315.00	0.988	274.00	0.537

DBS CRP, CRP measured in dried blood spot eluate; sCRP, salivary CRP; SES, socioeconomic status, calculated as sum of scores for parental education of both parents (4 = 12-13 years of schooling; 3 = 10 years of schooling; 2 = 9 years of schooling; 1 = < 9 years of schooling) and family income (6-levels: < 1000 Euro/month to > 5000 Euro/month) (sum-index, theoretical range: 3–14); BMI, body mass index. Sample size: Age: n = 87, BMI: n = 87, Sex: n = 45 (girls) n = 42 (boys), Medication intake: n = 8 (yes), n = 79 (no). **p < 0.01.

### 4.3 Bivariate Correlation of CRP Levels in DBS Eluate and Saliva

**Figure 1:**
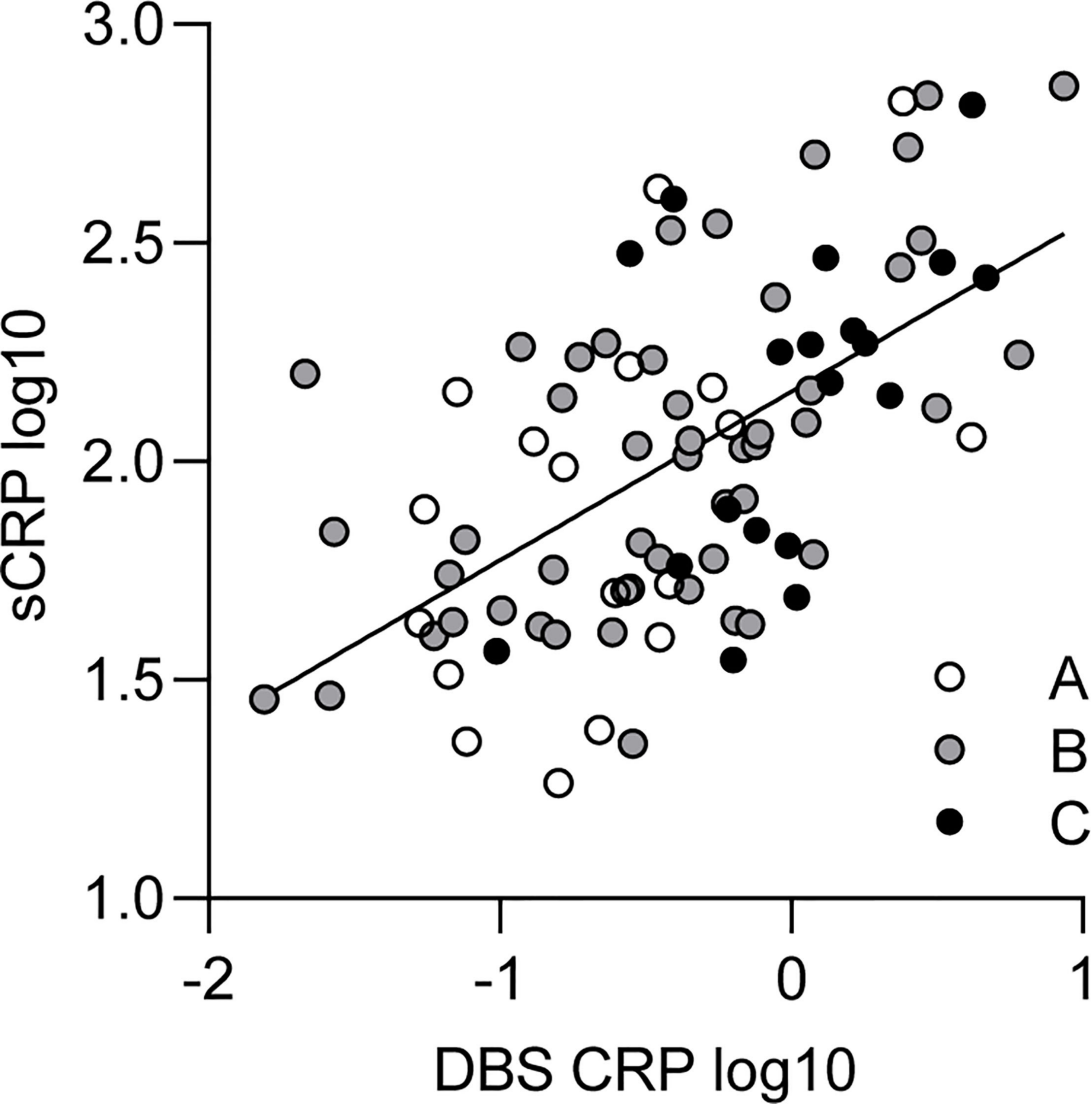
Relationship between DBS CRP and sCRP. White, gray, and black dots represent individual data points and their assignment to the respective BMI percentile range group (A: ≤ 25, *n* = 19; B: 26–74, *n* = 49; C: ≥ 75, *n* = 19). The solid line indicates the regression equation (*y* = 0.386*x + 2.160) of a simple linear regression calculated for the total sample [*R^2^* = 0.35; *F*(1, 85) = 45.74, *p* < 0.001]. DBS CRP, CRP measured in dried blood spot eluate; sCRP, salivary CRP; BMI, body mass index.

Pearson correlation of DBS CRP and sCRP revealed a significant association between both measures (*r* = 0.59, *p* < 0.001) (**Table 3**). Controlling for a covariate effect of BMI *via* partial correlation provided a comparable outcome (*r_p_* = 0.55, *p* < 0.001). In order to investigate whether the association of DBS CRP and sCRP differed between BMI-dependent subgroups, the sample was split into three BMI percentile range groups (A: ≤ 25, B: 26–74, C: ≥ 75). In all subgroups, DBS CRP and sCRP levels were significantly associated. Although the correlation coefficient of group B was highest (*r* = 0.61), calculating the *z*-scores of the differences between the correlations of group A and B/B and C revealed no significant outcome (**Table 3**): The correlation of DBS CRP with sCRP was equally high in all three BMI groups. Data are illustrated in **Figure 1**.

**Table 3:** Correlations of DBS CRP and sCRP levels in total and grouped by BMI percentiles.

Sample		Correlation of DBS CRP (log10) and sCRP (log10)				
	*n*	*r*		*p*		
Total	87	0.59		<0.001**		
Split by BMI (percentile)		***r***	***p***	***z_r_***	***Z_Difference_ to B***	***p***
A (≤25)	19	0.51	0.027*	0.56	−0.51	0.612
B (26–74)	49	0.61	<0.001**	0.71		
C (≥75)	19	0.53	0.020*	0.59	−0.41	0.682

DBS CRP, CRP measured in dried blood spot eluate; sCRP, salivary CRP; BMI, body mass index; A, BMI subgroup with percentile range ≤ 25; B, BMI subgroup with percentile range 26–74; C, BMI subgroup with percentile range ≥ 75; Z, z-scores of the differences between the correlations of the respective BMI subgroup and BMI subgroup B. *p < 0.05, **p < 0.01.

### 4.4 Comparison of Mean CRP Values per BMI Subgroup

**Figure 2:**
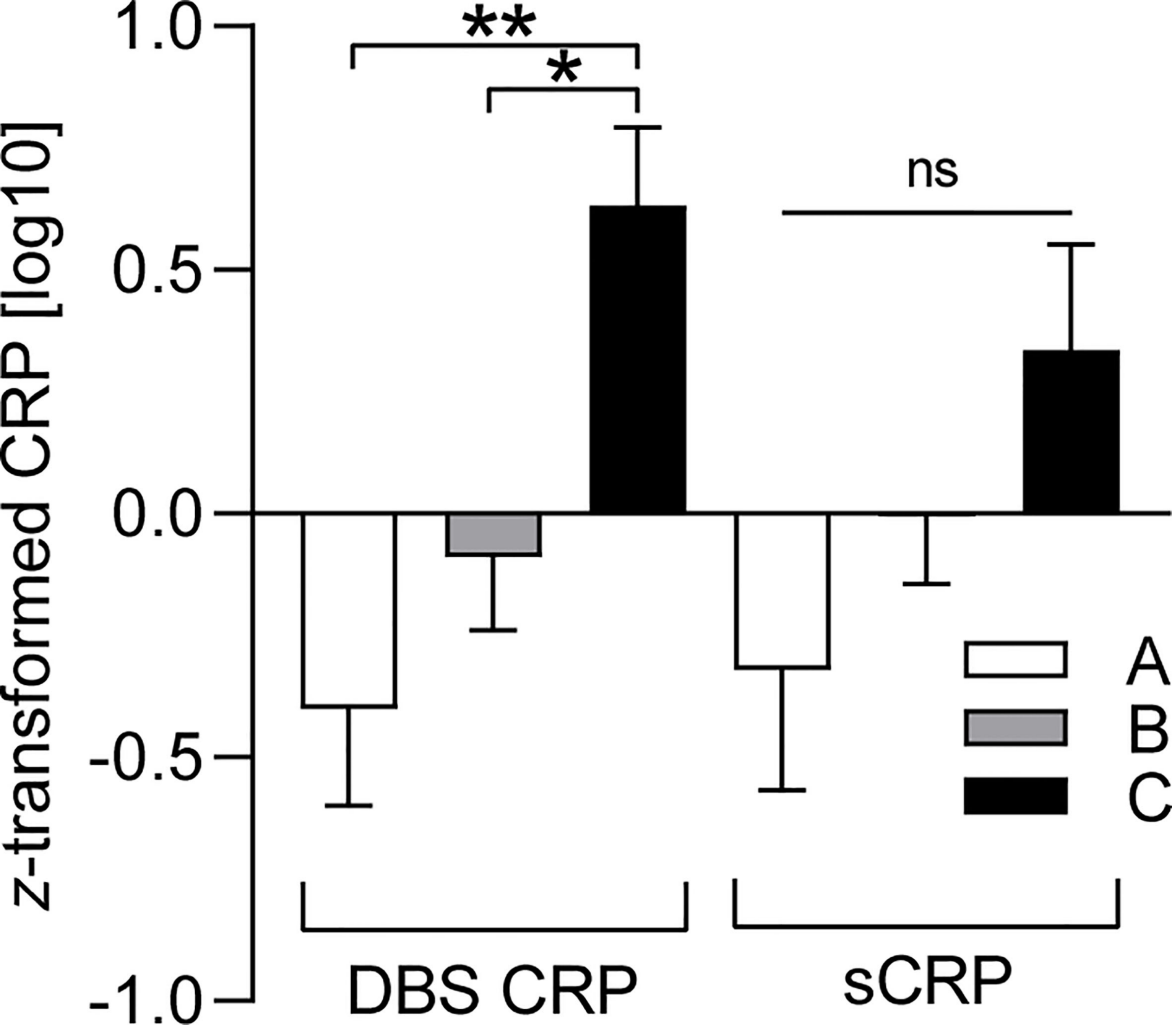
BMI-dependent distribution characteristics of DBS CRP and sCRP. DBS CRP (log10) and sCRP (log10) mean values per BMI percentile range group (A: ≤ 25, *n* = 19; B: 26–74, *n* = 49; C: ≥ 75, *n* = 19) were *z*-transformed for presentation purposes. One-way ANOVA followed by post-hoc testing revealed significant differences between the mean values of DBS CRP (group A and C: **p = 0.003, group B and C: *p = 0.017), but not those of sCRP (ns, not significant). Error bars: standard error of the mean. DBS CRP, CRP measured in dried blood spot eluate; sCRP, salivary CRP; BMI, body mass index.

Since the BMI was found to be associated with CRP levels (**Table 2**), we finally investigated whether the three BMI percentile range groups could be represented by different mean levels of DBS CRP and sCRP. One-way ANOVA reported a significant difference between the BMI groups regarding mean DBS CRP levels [*F*(2, 84) = 6.19, *p* = 0.003, with a medium effect size: *η* ^2^ = 0.13], but not for salivary CRP levels—even if the *η* ^2^ = 0.05 small effect pointed in the same direction (**Table 4**). Post-hoc testing (Hochberg’s GT2) revealed significant differences in mean DBS CRP levels between BMI group A and C (i.e., percentile range ≤ 25 vs. ≥ 75: *p* = 0.003) and between group B and C (percentile range 26–74 vs. ≥ 75: *p* = 0.017; group A vs group B: *p* = 0.531 ns): Higher BMI values were associated with higher CRP levels. *Z*-transformed data are presented in **Figure 2**.

**Table 4:** Descriptive statistics and ANOVA of DBS CRP and sCRP levels grouped by BMI.

BMI group (percentile)	DBS CRP (log10)			sCRP (log10)		
	*n*	*mean*	*SD*	*n*	*mean*	*SD*
A (≤25)	19	−0.61	0.51	19	1.89	0.41
B (26–74)	49	−0.43	0.62	49	2.01	0.38
C (≥75)	19	0.00	0.42	19	2.15	0.37
	***F (df, df)***	***p***	***η*** ^2^	***F (df, df)***	***p***	***η*** ^2^
ANOVA	6.19 (2, 84)	0.003**	0.13	2.10 (2, 84)	0.129	0.05

DBS CRP, CRP measured in dried blood spot eluate; sCRP, salivary CRP; BMI, body mass index; A, BMI subgroup with percentile range ≤ 25; B, BMI subgroup with percentile range 26–74; C, BMI subgroup with percentile range ≥ 75. **p < 0.01.

## 5 Discussion

The determination of CRP as a marker of systemic inflammatory processes is of great interest not only in clinical practice but also for a growing number of biobehavioral studies. Since the latter often require minimally invasive sampling methods, especially when young study participants are involved, we aimed to directly compare the measurement of CRP in eluate from dried blood spots and in saliva, both collected from healthy adolescents.

First, we found a significant, moderate to strong association of DBS CRP with sCRP in our sample (*r* = 0.59). Another direct comparison of DBS CRP and sCRP has been performed in the context of a stress test with adult African-Americans by Goetz and Lucas (39). In contrast to our findings, they only observed a modest, non-significant association between both measurements at baseline (*r* = 0.18) and a stronger, yet still moderate correlation of baseline DBS CRP with sCRP levels measured 1 h after the stressor task (*r* = 0.26). Overall, baseline CRP levels reported by Goetz and Lucas were higher than our mean values (DBS CRP T1 = 1.84 mg/L, sCRP T1 = 2,206.41 pg/ml) (39). These divergent outcomes might be due to the different age and/or cultural backgrounds of the study cohorts: although a similar sample size was investigated in both studies, our subjects were adolescents randomly sampled from the German population. In addition, differences in specimen collection, storage, and analysis as well as in data processing could affect the respective outcome. It is important to note that CRP levels measured in DBS eluate depend on the size of the punched blood spot and on the amount of fluid used for elution and do not directly correspond to serum CRP levels. Consequently, we cannot compare our absolute DBS CRP concentrations to those reported by other studies. Regarding sCRP baseline levels, literature reports on varying values with often wide ranges in healthy adults, e.g., mean (SEM) = 2,995 (573) pg/ml (30), 285 pg/ml (31), or 105 pg/ml (49). Mean sCRP values determined in young people also vary, but there are studies that, similar to our outcome, report lower levels in adolescents (80 pg/ml, mean age = 14 years) (33) and children (37 pg/ml, mean age 8 years, normal weight) (50).

An interesting aspect regarding DBS CRP and sCRP is its stress-reactivity kinetics, which Goetz and Lucas investigated (39). They report that sCRP, but not DBS CRP levels increased significantly from baseline to 1 h post stressor task performance, and hypothesize that DBS CRP was assessed too late to capture a peak response, since changes in CRP are considered to be observed more rapidly in blood than in saliva (39). In our study, participants attended a 2-h data collection session, including neuropsychological testing, interviews, and questionnaires, immediately before providing blood and saliva samples. Even if acute stress tests are not performed in this session, we cannot exclude that parts of it caused individually different levels of stress in our subjects. This, in turn, might have induced changes in CRP levels, which would have been captured differently by DBS CRP and sCRP at the moment of sample collection, due to their different stress reactivity kinetics. However, even baseline measurements at the beginning of a test session could be influenced by such distinct temporal reactivity, if induced by stressors occurring beforehand. Accordingly, Goetz and Lucas conclude that this difference in kinetics between DBS CRP and sCRP might partly explain a lack of strong associations between both measurements (39). In addition to this potential reason why we found that DBS CRP and sCRP were significantly correlated, but shared only 35% of common variance, several other aspects have also been discussed in studies comparing saliva with whole blood CRP.

First, the stability of CRP during storage has to be considered, as degradation after collection would bias the measurements. Brindle et al. (29) have investigated the stability of DBS CRP under various conditions. They suggest CRP in DBS to be stable at ambient temperature (21°C) for up to 1 week, and that stability of DBS stored at −20°C decreases after 1 year of storage (29). In the present study, all DBS samples were dried overnight at ambient temperature, then stored at −80°C, and measured within 1 year, so that storage conditions are unlikely to have caused significant degradation of CRP in our DBS samples. Salivary CRP levels have been shown to remain constant for up to 8 h after collection at room temperature before freezing at −20°C (30). Our saliva samples were frozen at −80°C within 0.5 h after collection, making a decline in sCRP before freezing unlikely as well. All sCRP samples were measured within 1 year, yet we cannot exclude that CRP levels in frozen saliva decline within this period, since we are not aware of published data on long-term stability of sCRP.

Second, levels of sCRP and other salivary biomarkers have been proven to vary during the day (32, 51–53), with a peak at awakening and lower levels during daytime (51). Such diurnal variation might have influenced the reliability of sCRP levels measured in our study, since sampling took place at different times of the day. However, data presented by Izawa et al. (51) indicates that the variation of sCRP levels between 10.00 a.m. and 17.00 p.m., when our sample collections were performed, was minimal. Mills et al. (54) showed that blood CRP levels of healthy adults do not change significantly within 24 h, so we conclude that diurnal variation effects are rather unlikely to have affected our outcome.

Lastly, effects of the oral cavity environment on sCRP levels have to be considered. CRP is assumed to enter saliva from systemic circulation *via* gingival crevicular fluid (GCF) (32, 36). However, local elevation of CRP levels induced by poor oral hygiene, oral inflammation, or diseases (38) could influence sCRP levels and thus weaken their association with blood CRP concentrations, especially when baseline systemic CRP levels are low (36). We controlled for oral health status in our sample *via* self-reports and excluded subjects who reported acute oral inflammation, injury, or diseases. Still, we cannot completely rule out cases with oral cavity issues, which we were not aware of.

In addition to these potential confounders of sCRP concentrations, both salivary and blood CRP levels could be influenced by other factors apart from systemic inflammatory conditions. We hence controlled for age, sex assigned at birth, regular medication intake during the past 6 months prior to DBS and saliva sampling, SES, and BMI [calculated as percentile according to Kromeyer-Hauschild et al. (47)], yet only the latter correlated significantly and positively with both DBS CRP (*r* = 0.40) and sCRP (*r* = 0.28) levels. This finding is in line with published literature, since BMI is a correlate of systemic inflammation (30) and is known to be positively associated with blood CRP levels (5, 40). Salivary CRP has also been shown to be significantly higher in obese than in normal weight children (50, 55), and to discriminate between lower and higher BMI in healthy adults when grouped in low sCRP vs. high sCRP (30). However, in the latter study, no significant correlation between BMI and the continuous sCRP measurement was detected (30), and Wettero et al. (52) did not confirm a significant association between sCRP and BMI in middle-aged participants. To further evaluate the validity of both the correlation between DBS CRP and sCRP and each measurement itself, we split our sample into three BMI percentile range groups: A: ≤ 25, B: 26–74, C: ≥ 75. Kromeyer-Hauschild et al. (47), who provide a BMI reference dataset for children and adolescents in Germany, recommend to refer to the 90th and 97th percentile as cutoff points for the definition of overweight and obesity, and to the 10th and 3rd percentile to define underweight and pronounced underweight (47). However, we decided to split our sample into the three groups described above, since we originally recruited the mothers of our study participants, generating a random sample of adolescents regarding their BMI, and did not seek to investigate body weight-related questions as a priority. This applies to many other biobehavioral studies interested in the role of inflammation in psychiatric disorders, or other fields of research. A second reason for the chosen percentile ranges was that only one girl was assigned to the 97th percentile in our sample, and eight adolescents to percentiles between 90 and 97 (five girls, three boys). Percentiles between the 10th and 3rd were calculated for four participants (two girls, two boys) and another four adolescents were assigned to the 3rd percentile or lower (two girls, two boys), so that defining percentile groups >90 and <10 would have resulted in too small sizes for statistical analysis (*n* < 10). We first computed the correlation of DBS CRP with sCRP per group and found that the associations of both measurements were comparably moderate to strong and significant in all three BMI groups (*r* = 0.51, *r* = 0.61, and *r* = 0.53 for groups A, B, and C, respectively). In practical terms, these data indicate that sCRP could be used as alternative measurement to DBS CRP regardless of body weight distribution within the study sample. We further compared the mean CRP values per BMI subgroup and measurement method and found a progressive increase in mean levels of both DBS CRP (medium effect) and sCRP (small effect) with increasing BMI range. This finding was expected, given the positive association of CRP with BMI, and is in line with published evidence that CRP levels are increased in the presence of subacute inflammation due to overweight or obesity (5, 40), whereas underweight has been associated with lower CRP in healthy adults compared to normal weight (56). However, differences between our BMI subgroups were only statistically significant for DBS CRP, but not for sCRP mean values. More specifically, the DBS CRP mean value of BMI group C (≥75) was significantly different from the two other group means, whereas groups A and B were not discriminated by DBS CRP average values. Loprinzi et al. (56) compared CRP levels in both underweight and overweight/obesity to normal weight and reported on a robust significant increase in CRP levels in overweight subjects and for all obesity classes, whereas CRP values in underweight were only stated to be significantly lower for the second of three tertiles (56). Less pronounced differences between under- and normal weight, combined with the fact that our BMI subgroup A was not restricted to BMI percentiles defining underweight (47) but comprised percentiles up to 25, might explain a lack of significance regarding the difference between mean DBS CRP of group A and B. On the other hand, since BMI group C was not restricted to percentiles classifying overweight/obesity (47) either, but included all subjects with percentiles greater than 75, it is noteworthy that DBS CRP mean values were still capable of significantly discriminating between BMI group B and C. Overall, these findings suggest that the criterion validity of DBS CRP is more pronounced than that of sCRP. However, adolescents with normal weight could still be distinguished from those that are classified as overweight or obese by sCRP mean levels (50, 55) if a greater sample size was analyzed.

Our effective sample size was significantly reduced due to several reasons, yet it is worth mentioning that 13 adolescents refused to provide blood for DBS, whereas only two participants did not agree in saliva sample collection. This distinct acceptance of sampling methods could have a significant impact on biobehavioral studies investigating small cohorts. Apart from the sample size, the fact that our cohort was derived from the general population could have limited the strength of the presented associations. Including individuals with clinical signs of systemic inflammation in future studies would provide a wider measuring range, which is required to further characterize the association between DBS CRP and sCRP. Another limitation of our study was that no other marker of inflammation, such as IL-6, IL-1β or TNF, was assessed, which would allow a more profound evaluation of the association between DBS and saliva measurements. However, to our knowledge, a highly sensitive method for the analysis of inflammatory cytokines from DBS has only been established recently (57) and could be applied in future research. Methodologically, determining blood contamination in saliva and establishing a detailed oral health profile including dental examination should be considered in future studies, as well as measuring salivary flow rate and total protein content for normalization of CRP values. Finally, we did not measure CRP in whole blood, which is still the gold standard for assessing CRP, since venipuncture was not feasible in our study. Future studies comparing intra-individual measurements of DBS, saliva and whole blood CRP could provide additional insight in the validity of both non-invasive approaches for different samples and research questions.

## 6 Conclusion

Since the assessment of CRP as a marker of inflammation is of growing interest in biobehavioral studies with young people, the present work aimed to directly compare two non-invasive approaches of CRP measurement in healthy adolescents. The significant correlation of DBS CRP with sCRP was independent of the investigated BMI range groups, yet BMI-dependent distinction was only provided by DBS CRP mean values. Overall, our results suggest that although DBS CRP is likely to reflect systemic inflammation more precisely, sCRP can be alternatively determined in studies with adolescents when conditions require it, given the oral health status is assessed.

## Data Availability Statement

The datasets presented in this article are not readily available because the raw data supporting the conclusions of this article are subject to data protection regulations, but can be made available on individual request. Requests to access the datasets should be directed to Dr. Anna Eichler, Anna.Eichler@uk-erlangen.de.

## Ethics Statement

The studies involving human participants were reviewed and approved by Ethik-Kommission der Friedrich-Alexander Universität Erlangen-Nürnberg (FAU) (353_18B). Written informed consent to participate in this study was provided by the participants’ legal guardian/next of kin.

## Author Contributions

Conceptualization: NR, PF, MB, JK, AE, GM, IMAC-Mind-Consortium, and OK. Methodology: A-CP, NR, PF, MB, JK, AE, GM, and OK. Investigation: A-CP, JM, and AE. Data curation: A-CP, JM, and AE. Writing—original draft preparation: A-CP and OK. Writing—review and editing: JM, NR, PF, MB, JK, AE, GM, and IMAC-Mind-Consortium. Visualization: A-CP and AE. Validation: A-CP, AE, and OK. Supervision: NR, AE, and OK. Project administration: PF, MB, JK, AE, GM, and OK. Funding acquisition: PF, MB, JK, AE, GM, OK, and IMAC-Mind-Consortium. All authors contributed to the article and approved the submitted version.

## Funding

This project was partly funded by the Federal Ministry of Education and Research (IMAC-Mind: Improving Mental Health and Reducing Addiction in Childhood and Adolescence through Mindfulness: Mechanisms, Prevention and Treatment; 01GL1745B subproject TP1), the Else Kröner-Fresenius-Stiftung (2019_A45, grant to AE), and the Universitätsbund Erlangen-Nürnberg (no grant number, grant to AE).

## Conflict of Interest

The authors declare that the research was conducted in the absence of any commercial or financial relationships that could be construed as a potential conflict of interest.

## Publisher’s Note

All claims expressed in this article are solely those of the authors and do not necessarily represent those of their affiliated organizations, or those of the publisher, the editors and the reviewers. Any product that may be evaluated in this article, or claim that may be made by its manufacturer, is not guaranteed or endorsed by the publisher.
